# Diagnostic Performance of Cystatin C in the Early Detection of Diabetic Kidney Disease at the University of Nigeria Teaching Hospital, Ituku-Ozalla

**DOI:** 10.7759/cureus.72230

**Published:** 2024-10-23

**Authors:** Ndubuisi V Nwanonenyi, Chuba k Ijoma, Ejike Arodiwe, Maris-Stella I Nwanonenyi, Chidinma Nebo

**Affiliations:** 1 Nephrology, University of Nigeria Teaching Hospital, Enugu, NGA; 2 Internal Medicine, University of Nigeria Teaching Hospital, Enugu, NGA; 3 Nephrology, Enugu State University Teaching Hospital, Enugu, NGA

**Keywords:** albumin creatinine ratio, chronic kidney disease, cystatin c, diabetes mellitus, diabetic kidney disease, end stage kidney stage renal disease, estimated glomerular filtration rate, modification of disease in renal disease, receiver operating curve, urine albumin excretion rate

## Abstract

Introduction: There is an increase in the prevalence of diabetes mellitus (DM) globally. Individuals with diabetes mellitus are at higher risk of impairment of kidney function. This study evaluated the diagnostic performance of Cystatin C in the early detection of diabetic kidney disease (DKD).

Methods: Across a sectional analytical study of 300 participants (200 study group and 100 control group). A relevant clinical history was obtained, and a physical examination was carried out. Venous blood was collected to assay for serum creatinine, serum albumin, serum cystatin C, serum urea, fasting blood glucose, and urine for the quantification of urine albumin excretion rate.

Results: The median age of the study group versus the control group was 62.50 for DM with proteinuria, 60.00 for DM without proteinuria, and 60.00 years for the control group (F = 3.524, p = 0.172). The laboratory parameters that were higher in the study group compared to the control group were FBG (141.0, 130, vs. 104 mg/dl, F = 68.456, p = <0.001), serum creatinine (109.0, 88.5, vs. 105.0 umol/l, F = 35.50, p = <0.001), serum cystatin C (1.24, 1.11, vs. 0.84 mg/L, F = 59.27, p = <0.001), and urine albumin excretion (230.0, 102.0, vs. 30.0 mg, F = 128.62, p = <0.001). The diagnostic performance of cystatin C using MDRD and cystatin C eGFR <60ml/min/1.73m2 was 13% and 23%, respectively, for the study group without proteinuria. Also, when the diagnostic efficiency of the variables was compared using ROC, the AUC of creatinine eGFR (MDRD) was less than that of cystatin C eGFR between the cut-off levels of 30 mg and 300 mg of urine albumin excretion. Cystatin C eGFR had a strong negative correlation with urine albumin excretion when compared to creatinine eGFR (MDRD).

Conclusion: This study showed the diagnostic performance of serum cystatin C in the early detection of DKD and that cystatin C-derived eGFR is more sensitive than serum creatinine-derived eGFR in detecting DKD early in people with DM.

## Introduction

Diabetic kidney disease (DKD) is a syndrome marked by the presence of macroalbuminuria [1], a slow and progressive decline in glomerular filtration rate (GFR), elevated blood pressure, and cardiovascular mortality [2]. Worldwide, DM is the most common cause of chronic kidney disease [3]. Diabetic kidney disease is more frequent in African Americans, Asian Americans, and Native Americans. Progressive diabetic nephropathy is also more frequent in Caucasian patients with type 1 than type 2 DM, though it has a higher prevalence in type 2 diabetic patients because this type of DM is more prevalent [2].

Since the 1950s, kidney disease has been clearly recognized as a common complication of diabetes mellitus (DM), with as many as 50% of patients with DM of more than 20 years’ duration developing this complication. The development of assays for the detection of microalbuminuria (MA) in the 1960s revolutionized diabetes management. Microalbuminuria, defined as urinary albumin excretion rate (UAE) 30-300 mg/d, is the earliest and most used clinical index of DKD [4]. Microalbuminuria is independently associated with cardiovascular risk in diabetic patients, due in part to its role as an indicator of widespread microvascular disease and of underlying renal disease [4]. Many studies have since indicated that a reduction of UAE in type 2 diabetic patients reflects renal and cardiovascular risk reduction [4,5]. Early detection of poor glomerular and tubular function can be achieved with biomarkers of glomerular and tubular dysfunction. Biomarkers such as cystatin C can serve to evaluate kidney function in people with DKD even when microalbuminuria is absent [6].

Diabetic kidney disease is defined as albuminuria (albumin excretion rate > 300 mg/24 h) and declining renal function in a patient with known DM in the absence of urinary tract infection or any other renal disease [4]. Although UAE remains a crucial tool for risk stratification and monitoring disease progression [4], several factors have called into question its sensitivity and specificity [4].

The presence of MA was originally thought to be predictive of future overt DKD in 80% of patients [4]. However, more recent evidence suggests that only around 30% of microalbuminuric patients progress to overt nephropathy after 10 years of follow-up [4,7]. It has also been shown that advanced structural alterations in the glomerular basement membrane may already have occurred by the time MA becomes clinically evident [4,8]. In addition, there is evidence that a significant proportion of patients with MA can revert to normoalbuminuria [4,9] and the concept of normoalbuminuric DKD is well-documented, reflecting the fact that patients with diabetes can demonstrate a reduction in glomerular filtration rate without progressing from normo-to MA [4,8,10].

Serum creatinine (SCr) level is the most used marker for assessing renal impairment. It is found to be relatively insensitive for detecting early renal impairment in DM, especially in hyperfiltration status [11]. Since DM causes glomerular and tubular changes, tubular marker proteins may be used to detect early renal damage associated with DKD. Therefore, there is a need to identify and investigate alternative biomarkers [4] such as cystatin C for the earlier prediction and diagnosis of DKD.

Few studies have been done on the use of cystatin C as an early diagnostic marker of DKD in Nigeria. Godwill O et al. [12] in 2013 did a one-year retrospective study on 50 black patients with type 2 DM recruited from the metabolic clinic of the University of Port-Harcourt Teaching Hospital. Their study suggested that cystatin C may be more effective than creatinine in detecting early renal impairment in patients with type 2 diabetes [12]. Also, DM has emerged as a major and global public health challenge, reaching epidemic proportions [13]. The consequent DKD and poor outcomes, which are not detected by the traditional DKD [13] tests such as MA and SCr, call for a more inclusive approach in the early detection of patients who are at risk of developing DKD.

Microalbuminuria (MA) is the earliest and most used clinical index of DKD; however, its sensitivity and specificity for early disease detection are limited [4]. Not all patients with MA progress to overt DKD; nonalbuminuric DKD is common, and the risk associated with MA is elevated even at levels below currently accepted diagnostic thresholds [4]. There is therefore a need for an alternative biomarker (cystatin C) allowing early identification of “at-risk” individuals [4] and the severity of disease in patients already with DKD.

## Materials and methods

Study area

This research was carried out at nephrology and diabetic clinics in the medical outpatient department, the general outpatient department, and the medical wards of the University of Nigeria Teaching Hospital (UNTH), Ituku-Ozalla, and it is managed by consultants specializing in nephrology and endocrinology, senior registrars, registrars, house officers, and medical officers.

Study population

The study population was made up of patients with DM attending both the endocrinology and nephrology clinics of the Medical Outpatient Department (MOPD) and General Outpatient Department (GOPD), as well as those on admission in the medical wards at UNTH, Ituku-Ozalla, Enugu State. All case subjects have diabetes, impaired eGFR (MDRD <60 ml/min/1.73 m^2^), normal kidney function, hypertension, or no hypertension, while the controls were apparently healthy subjects.

Study design

This was a hospital-based cross-sectional analytical study. A consecutive study population that met the inclusion criteria was recruited for the study.

Data collection

An interviewer-administered questionnaire was used to obtain socio-demographic data and medical history. This was augmented with patients’ case notes to minimize recall bias as much as possible. Participants who were matched for age and sex with participants in the study group were recruited into the control arm.

Predesigned questionnaires, indicating the following:

(a) Biodata, (b) diagnosis (indicating type of DM) (c) co-morbidity (CKD including etiology) [13], (d) drug history: oral hypoglycemic agent, insulin, anti-hypertensive (e) dialysis, including duration (f) history of blood transfusion. (g) social history: cigarette smoking, alcohol (g) family history of DM (h) examinations, including anthropometry, were administered to both the study groups and the controls.

Blood samples were collected early in the morning (9:00-11:00) to minimize the effect of diurnal variation on the results, and the following biochemical investigation (serum creatinine, cystatin C, albumin, urea, FBG, urinalysis (dipsticks), urine albumin, and creatinine were tested and analyzed.

Data analysis

Data were analyzed using IBM Corp. Released 2015. IBM SPSS Statistics for Windows, Version 23.0. Armonk, NY: IBM Corp., and results were presented as tables and graphs where appropriate. The diagnostic performance of serum cystatin C was determined using ROC analysis. Continuous independent variables (study and control group) were tested for normality of distribution using the Shapiro-Wilk test. Continuous variables were presented as means and standard deviation for normally distributed data and median with interquartile range for skewed data. Categorical variables were presented as frequency and percentages. Chi-square or Fisher’s exact was used to compare categorical variables [13]. Correlations between eGFR (MDRD/cystatin C-based) [13] and urine albumin excretion (UAE) were determined by Spearman’s correlation test. An ANOVA test for skewed data (Kruskal-Wallis test) was conducted to check whether there was any difference in the median values of the analytes across the participant groups. ROC analysis was used to assess and compare the diagnostic efficiency of eGFR (MDRD/cystatin C). A p-value of <0.05 was considered statistically significant [13].

Ethical consideration

This study was approved by the Ethics and Research Committee of UNTH Ituku/Ozalla [13], with referral number NHREC/05/01/2008B-FWA00002458-1RB00002323. All participants signed written informed consent before enrollment in the study [13], and there was no financial burden on the participants. Study participants were given the freedom to withdraw from the study if they wished. All the information obtained from the participants was kept confidential, and samples were stored for 90 days, after which they were appropriately discarded. The results of the investigations were made available to the participants and were used for their management where necessary.

## Results

The general characteristics of the participants are shown in Table 1. In this study, the participants were divided into three groups or arms, comprising diabetics with proteinuria, diabetics without proteinuria, and those without diabetes (controls). A total of 200 diabetic patients (100 with proteinuria and another 100 without proteinuria) and 100 age- and sex-matched controls that satisfied the inclusion criteria were recruited into the study with a median age of 62 and 60.

**Table 1:** Demographic and clinical characteristics of the study population X2: Chi-square; F: ANOVA/Kruskal-Wallis test; a Kruskal-Wallis test; b: Chi-square test; c: ANOVA test; *: test statistic is significant at 0.05 level; BMI: Body mass index (Kg/M2); FBS: Fasting blood sugar (Mg/dl); SBP: Systolic blood pressure (mmHg); DSB: Diastolic blood pressure (mmHg).

Variable		DM with proteinuria n = 100 (%)	DM without proteinuria n = 100 (%)	Control n = 100 (%)	X^2^/F	p-value
Age (years)	< 30	0 (0.0)	2 (2.0)	7 (7.0)	3.524^a^	0.172
	31 – 45	9 (9.0)	15 (15.0)	13 (13.0)		
	46 – 60	33 (33.0)	37 (37.0)	37 (37.0)		
	61 – 75	55 (55.0)	43 (43.0)	42 (42.0)		
	> 75	3 (3.0)	3 (3.0)	1 (1.0)		
	Median	62.50	60.00	60.00		
	Range	(32 – 84)	(26 – 85)	(25 – 77)		
Gender	Male	50 (50.0)	50 (50.0)	50 (50.0)	0.080^b^	0.961
	Female	50 (50.0)	50 (50.0)	50 (50.0)		
Education	No formal education	12 (12.0)	11 (11.0)	2 (2.0)	74.122^b^	<0.001*
	Primary school	36 (36.0)	36 (36.0)	5 (5.0)		
	Secondary school	25 (25.0)	28 (28.0)	17 (17.0)		
	Tertiary	27 (27.0)	25 (25.0)	76 (76.0)		
BMI	Mean	28.31	28.31	28.94	0.612^c^	0.543
	SD	5.72	4.63	5.20		
FBS	Median	141.0	130.0	104.0	68.456^a^	<0.001*
	Range	(55 – 459)	(60 – 600)	(67 – 126)		
SBP	Median	136.5	130	120	44.139^a^	<0.001*
	Range	(90 – 188)	(96 – 170)	(99 – 144)		
DBP	Median	80.0	80.0	74	33.302^a^	<0.001*
	Range	(58 – 100)	(60 – 190)	(55 – 89)		

The distribution of urine albumin excretion (UAE), creatinine, and cystatin C showed that increasing UAE (230.0, p = <0.001) was associated with increasing levels of creatinine (109.0, p = <0.001) and cystatin C (1.24, p = <0.001) in diabetic patients when compared to the control group (Table 2). There is a statistically significant change in the level of cystatin C as one progresses from DM patients without proteinuria to the normal population (controls), thus revealing early changes in renal function among the groups.

**Table 2:** Distribution of UAE, cystatin C, and creatinine in the study population F: Kruskal-Wallis test; *: test statistic is significant at 0.05 level

Variable		DM with proteinuria n = 100 (%)	DM without proteinuria n = 100 (%)	Control n = 100 (%)	F	p-value
Urine Albumin Excretion (mg)	Median	230.0	102.0	30.0	128.62	<0.001*
	Range	(19 – 3255)	(15 – 85)	(3.4 – 215)		
Serum Creatinine (umol/L)	< 106 umol/L	50 (50.0%)	80 (81.0%)	63 (63.0%)		
	> 106 umol/L	50 (50.0%)	19 (19.0%)	37 (37.0%)		
	Median	109	88.5	102	35.50	<0.001*
	Range	(64 – 1547)	(61 – 135)	(63 – 184)		
Serum Cystatin C (mg/L)	< 0.64 (mg/L)	0 (0.0%)	14 (14.0%)	40 (40.0%)		
	0.64 – 1.12 (mg/L)	28 (28.0%)	37 (37.0%)	33 (33.0%)		
	> 1.12 (mg/L)	72 (72.0%)	49 (49.0%)	27 (27.0%)		
	Median	1.24	1.11	0.84	59.27	<0.001*

In the studied patient population, decreasing estimated GFR was associated with increasing cystatin C and creatinine levels as proteinuria worsens. However, there is a significant statistical difference in the median values of cystatin C-based estimated GFR when compared to that of creatinine-based estimated GFR (MDRD) (Table 3).

**Table 3:** Diagnostic abilities of creatinine eGFR (MDRD), cystatin C eGFR X2: Chi-square; *: test statistic is significant at 0.05 level

Variable		DM with proteinuria n = 100 (%)	DM without proteinuria n = 100 (%)	Control n = 100 (%)	X^2^	p-value
MDRD (60ml/min/1.73m^2^)	< 60	38 (38.0)	13 (13.0)	7 (7.0)	34.349	<0.001*
	>/= 60	62 (62.0)	87 (87.0)	93 (93.0)		
Cystatin C eGFR (60ml/min/1.73m^2^)	< 60	53 (53.0)	23 (23.0)	10 (10.0)	48.285	<0.001*
	>/= 60	47 (47.0)	77 (77.0)	90 (90.0)		

Also, the diagnostic abilities of creatinine-based (MDRD) GFR when compared to that of cystatin C revealed that cystatin C-based estimated GFR performed better. At an estimated GFR cutoff of >60 ml/min, cystatin C reflected more patients without proteinuria than creatinine. Therefore, MDRD underestimated GFR in DM patients without proteinuria (Table 4).

**Table 4:** Distribution of creatinine eGFR (MDRD) and cystatin C eGFR in the study population F: Kruskal-Wallis test; *: test statistic is significant at 0.05 level

Variable		DM with proteinuria n = 100 (%)	DM without proteinuria n = 100 (%)	Control n = 100 (%)	F	p-value
MDRD	Median	59.0	75.0	95.5	52.741	<0.001*
	Range	(6 – 110.0)	(13 – 145)	(31 – 186)		
Cystatin C eGFR	Median	70.5	89.7	91.5	79.984	<0.00*
	Range	(4.1 – 120.6)	(16.0 – 146.5)	(10 – 145)		

Also, when we looked at the distribution of serum cystatin C among DM patients without proteinuria, even though at cutoff >1.12, serum cystatin C was able to reveal a patient, this was statistically significant. It also showed more than 50% of the DM patients with proteinuria have deranged serum cystatin C (Table 5).

**Table 5:** Distribution of serum cystatin C in the study group with DM without proteinuria X2: Chi-square; *: test statistic is significant at 0.05 level

		Urine Albumin Excretion (mg)		X^2^	p-value
		< 30	30 – 299		
Serum cystatin C (umol/L)	= 1.12	14 (93.3)	36 (48.0)	10.404	0.001*
	> 1.12	1 (6.7)	39 (52.0)		
	Total	15 (100.0)	75 (100.0)		

**Figure 1:**
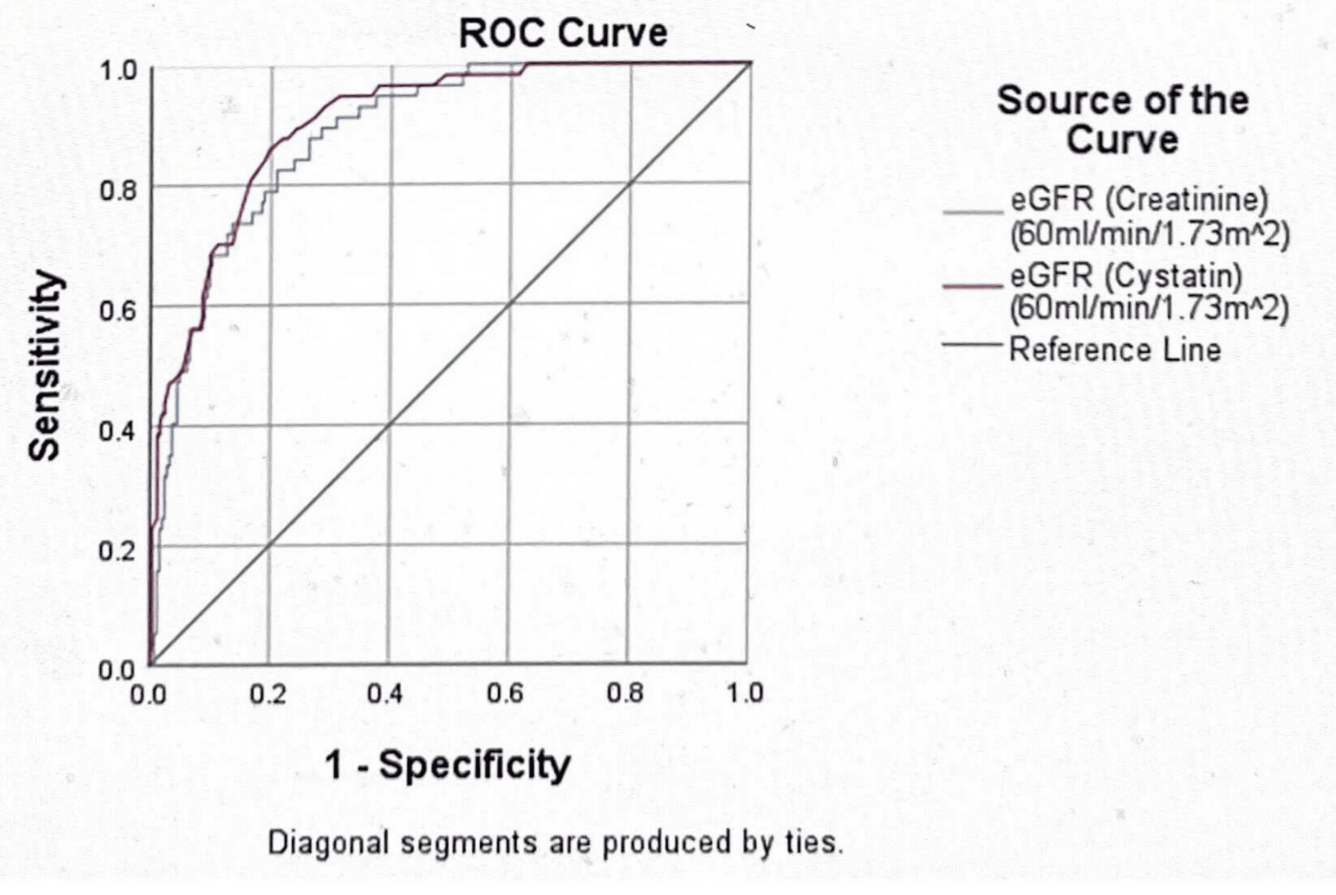
The ROC curve at the urine albumin excretion cut-off of 30 mg/dl

**Figure 2:**
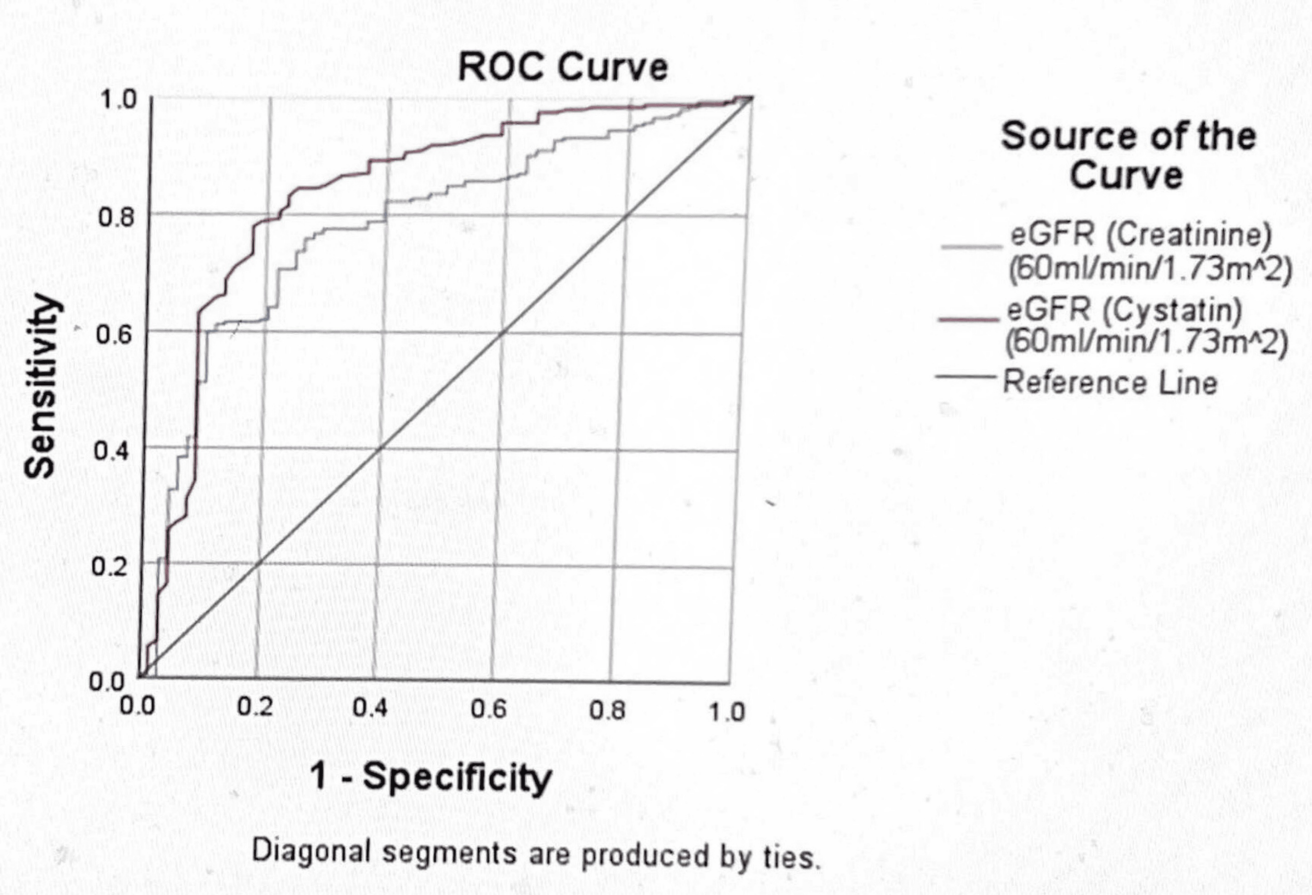
The ROC curve at the urine albumin excretion cut-off of 300 mg/dl

The AUC of creatinine eGFR (MDRD) was less than that of cystatin C eGFR between the cut-off levels of 30 mg and 300 mg of urine albumin excretion. Thus, reflecting a higher sensitivity of cystatin C eGFR than that of creatinine eGFR (MDRD) at micro-albuminuria and macro-albuminuria cut-off levels (Figures 1, 2).

## Discussion

Diabetic patients with DKD face a significantly higher risk of developing ESRD with increased mortality and morbidity due to cardiovascular disease [1]. Albuminuria is a tool widely used for risk assessment and progression analysis of DKD. An accepted gold standard with better sensitivity and specificity for the earlier diagnosis and assessment of disease progression of DKD is still not agreed upon [14]. Serum creatinine is the analyte typically used to estimate GFR in routine practice. Serum creatinine has poor sensitivity in the early stages of kidney dysfunction, as a significant decrease in GFR has usually occurred by the time serum levels begin to rise [1,15,16].

GFR estimated from serum cystatin C may more accurately reflect kidney function than GFR estimated using traditional serum creatinine-based methods [17] since the main factor influencing serum cystatin C levels in the blood is the rate at which it is filtered by the glomerulus [1], and it is more strongly associated with adverse health outcomes in community-based populations, the majority of whom are nondiabetic [17-19].

Four methods of evaluation (correlation with GFR, mean values of each variable in DM patients stratified by AUE values, ROC curves, and diagnostic efficiency) were used in our study [12,20]. We demonstrated that cystatin C is more sensitive than creatinine and creatinine-derived formulas in detecting early renal function impairment [12].

The correlation of cystatin C with GFR was stronger than the correlation with creatinine, or MDRD formula [20]. As previously reported in type 2 diabetes and observed in our study, the correlations between GFR and either creatinine or cystatin C were stronger in patients with decreased GFR compared to those with normal GFR [12]. The primary factor determining cystatin C levels in the blood is the rate at which it is filtered by the glomerulus, which is an excellent marker of GFR [1,16,21]. We categorized the study into DM with proteinuria and DM without proteinuria. When their eGFR is compared, it could be seen that serum cystatin C picked more patients with eGFR < 60 ml/min/1.73 m^2^ than serum creatinine [22]. This is like the findings by You Jeon et al. [22] in their study in South Korea. Other studies were able to link worsening eGFR and UAE or ACR to increased morbidity and mortality. De Boer IH et al. [17] demonstrated in their study that cystatin C and creatinine eGFR <60 ml/min/1.73 m2 and UACR or UAE > or = 30 mg/g were each associated with increased mortality. Sema Uslu et al. [23] also noted that the maximum diagnostic accuracy of serum cystatin C (90%) was significantly better than that of serum creatinine (77%), and concluded that cystatin C may be considered as an alternative and more accurate serum marker than serum creatinine or the Cockcroft and Gault estimated GFR in discriminating type 2 diabetic patients with reduced GFR from those with normal GFR [23]. 

We also found that in our study, serum cystatin C and urine albumin were found to be strongly positively correlated [23]. Though the DM group without proteinuria was found to have microalbuminuria, this is attributed to the method of selection, which was through urine dipstick. However, UAE was low, mainly amongst diabetics without proteinuria. A study by Oddoze et al. [24] reported no correlation between serum cystatin C and urine albumin. This could be due to the small sample size of 49 used in the study.

## Conclusions

In addition to renal function, multiple factors affect the level of serum cystatin C. It is evident from the study that serum cystatin C and cystatin C-derived eGFR performed better than serum creatinine and creatinine-derived eGFR in detecting DKD, especially in those asymptomatic for albuminuria. It also showed that serum cystatin C levels increased more in those with worsening proteinuria and eGFR. It also showed a significant association between serum cystatin C level, serum creatinine level, eGFR, and urine albumin excretion (UAE). Albuminuria and eGFR are independent risk factors for mortality among patients with DM. This study suggests that serum cystatin C may be more effective in detecting renal impairment in patients with DM than creatinine or creatinine-based formula. It also supports the need to investigate patients with diabetes for the presence of albuminuria with the aim of treating and preventing complications such as CVD and ESRD. Detecting early renal impairment using cystatin C would be highly beneficial in managing DM as it would enable the timely initiation of appropriate therapeutic measures, leading to improved treatment outcomes for this patient group.

## References

[1] Qamar A, Mahmood Ahmad T, Hayat A, Alamgir Khan M, Najam Ul Hasnat M, Rehman S (2017). Renal function assessment by cystatin C and creatinine based equations. Pak Armed Forces Med J.

[2] Gheith O, Farouk N, Nampoory N, Halim MA, Al-Otaibi T (2016). Diabetic kidney disease: world wide difference of prevalence and risk factors. J Nephropharmacol.

[3] Jha V, Garcia G, Iseki G (2013). Chronic kidney disease_ global dimension and perspectives. Lancet.

[4] Currie G, McKay G, Delles C (2014). Biomarkers in diabetic nephropathy: present and future. World J Diabetes.

[5] Araki S, Haneda M, Koya D (2007). Reduction in microalbuminuria as an integrated indicator for renal and cardiovascular risk reduction in patients with type 2 diabetes. Diabetes.

[6] Ameer S, Nabil AEK, Ahmed AS, El-Deen K, Barbary H (2014). Evaluation of serum cystatin C as an indicator of early renal function decline in type 2 diabetes. Meno Med Jr.

[7] Rossing P, Hougaard P, Parving HH (2005). Progression of microalbuminuria in type 1 diabetes: ten-year prospective observational study. Kidney Int.

[8] Perkins BA, Ficociello LH, Roshan B, Warram JH, Krolewski AS (2010). In patients with type 1 diabetes and new-onset microalbuminuria the development of advanced chronic kidney disease may not require progression to proteinuria. Kidney Int.

[9] Perkins BA, Ficociello LH, Silva KH, Finkelstein DM, Warram JH, Krolewski AS (2003). Regression of microalbuminuria in type 1 diabetes. New Eng Jr Med.

[10] Kramer HJ, Nguyen QD, Curhan G, Hsu CY (2003). Renal insufficiency in the absence of albuminuria and retinopathy among adults with type 2 diabetes mellitus. JAMA.

[11] Lin CH, Chang YC, Chuang LM (2016). Early detection of diabetic kidney disease: Present limitations and future perspectives. World J Diabetes.

[12] Godwill O, Ntuen N Use of serum cystatin C in assessment of early deterioration of renal function in type 2 diabetic industrial workers in Port Harcourt, Nigeria. Jr Den Med Sci.

[13] Soje Michael.O (2017). Serum Albumin and C-reactive Protein in Adult Nigerians with Chronic Kidney Disease in Federal Teaching Hospital Ido Ekiti.

[14] Macisaac RJ, Ekinci EI, Jerums G (2014). Markers of and risk factors for the development and progression of diabetic kidney disease. Am J Kidney Dis.

[15] Avinash S, Singh VP, Agarwal AK, Chatterjee S, Arya V (2015). Identification and stratification of diabetic kidney disease using serum cystatin C and serum creatinine based estimating equations in type 2 diabetes: A comparative analysis. Jr Asso Phys Ind.

[16] Al-Maqbali SRS, Mula-Abed WAS (2014). Comparison between three different equations for the estimation of glomerular filtration rate in Omani patients with type 2 diabetes mellitus. https://pmc.ncbi.nlm.nih.gov/articles/PMC3997536/.

[17] de Boer IH, Katz R, Cao JJ (2009). Cystatin C, albuminuria, and mortality among older adults with diabetes. Diabetes Care.

[18] Shlipak MG, Katz R, Sarnak MJ (2006). Cystatin C and prognosis for cardiovascular and kidney outcomes in elderly persons without chronic kidney disease. Ann Intern Med.

[19] Shlipak MG, Sarnak MJ, Katz R, Fried LF, Seliger SL, Newman AB, Siscovick DS SBC (2005). Cystatin C and the risk of death and cardiovascular events among elderly persons. N Engl J Med.

[20] Pucci L, Triscornia S, Lucchesi D (2007). Cystatin C and estimates of renal function: searching for a better measure of kidney function in diabetic patients. Clin Chem.

[21] Christensson AG, Grubb AO, Nilsson JA, Norrgren K, Sterner G, Sundkvist G (2004). Serum cystatin C advantageous compared with serum creatinine in the detection of mild but not severe diabetic nephropathy. J Intern Med.

[22] Jeon YK, Kim MR, Huh JE (2011). Cystatin C as an early biomarker of nephropathy in patients with type 2 diabetes. J Korean Med Sci.

[23] Ebaokncodcya SU (2005). Serum cystatin C and urinary enzymes as screening markers of renal dysfunction in diabetic patients. J Nephrol.

[24] Oddoze C, Morange S, Portugal H, Berland Y, Dussol B (2001). Cystatin C is not more sensitive than creatinine for detecting early renal impairment in patients with diabetes. Am J Kidney Dis.

